# An illusion of disownership over one’s own limb is associated with pain perception

**DOI:** 10.1038/s41598-023-29993-z

**Published:** 2023-03-01

**Authors:** Yuta Nishiyama, Chihiro Yamashita, Shusaku Nomura

**Affiliations:** Department of Information and Management Systems Engineering, Nagaoka University of Technology, Niigata, Japan

**Keywords:** Pain, Consciousness, Perception

## Abstract

Viewing one’s body and even a fake/virtual body experienced as one’s own has been suggested to modulate pain perception. However, what happens to pain perception when one’s own body part is felt as not belonging to one? We designed a paradigm to induce an illusory feeling of disownership regarding one’s limb, investigating whether the feeling affects pain threshold. Participants observed right-side images of their bodies from a third-person perspective via a head-mounted display in real-time. Following instructions, they moved their left hand while keeping their left elbow behind the upper body, so that the connection of their arm to the torso was not visible (test condition), or in front of it, so they could see the arm being part of them (control condition). Then, pain threshold was tested with a thermal stimulator. We found a significantly higher strength of disownership in the test condition than in the control condition. While there was no pain modulation within and between conditions, disownership ratings negatively correlated with pain-threshold changes, where the participants reporting explicit disownership showed lower pain-threshold changes than the others. The finding suggests that while multisensory disintegration had no modulatory effect, the individual sense of disownership was associated with pain perception.

## Introduction

Pain is a complex, conscious experience extending beyond the result of a simple bottom-up brain process that originates from noxious stimuli. Multiple brain regions can be engaged in top-down regulation to modulate the pain experience^1^. In particular, cognitive representations of the body activated by viewing “one’s own” body influence a set of brain areas activated by painful stimuli^2,3^. For instance, according to an experimental study with healthy participants, seeing one’s own hand produces lower subjective ratings of pain intensity and decreases brain responses elicited by nociceptive stimuli when compared with seeing an object or another person’s hand^4^. Similar effects of visual feedback have been reported in clinical populations as well. Phantom limb syndrome, the sensation that an amputated body part is still present, is often accompanied by pain in the affected part even though it no longer exists^5^. Mirror therapy has been used as a treatment for phantom limb syndrome^6^. Patients are presented with the mirror image of their intact limb at the location where the phantom limb is felt. Here, they feel as if their lost limb is still there, leading to the alleviation of phantom pain during and also after the training. This suggests that viewing a body that one perceives as “one’s own” can relieve pain on the part even if the viewed body is actually not one’s own.

The feeling that “my body is mine” is taken for granted in healthy populations. However, experimental manipulation by means of conflicting multimodal stimulation can temporarily induce the illusion of owning an external object resembling the human body that does not belong to you (for a review see Ref.^7^). In the rubber hand illusion, participants perceive a fake hand as part of their own body while watching the fake hand being touched synchronously with an unseen real hand^8,9^. Watching video images of one’s own body projected on a head-mounted display (HMD) generate an out-of-body illusion, or full-body illusion, in which participants wearing the HMD simultaneously receive visual and tactile stimulation of touch being away from each other^10–12^. Moreover, when participants observe a virtual world via an HMD, taking a first person perspective of the virtual body produces transformations in body ownership^13,14^.

It has been suggested that viewing the proxy body part—that feels as if it belongs to one’s own body—produces modulatory effects on pain perception as in the situation of looking at one’s own body (for a review see Ref.^15^), although there are inconsistent results^16^. One pioneering study on body ownership and pain perception using rigorously controlled experiments found no modulation of heat pain intensity, heat/cold pain thresholds nor temperature perception thresholds in the rubber hand illusion^16^. Although still controversial, subsequent studies have reported increases in pain thresholds. Hegedüs and colleagues demonstrated that both the rubber hand illusion and seeing one’s own hand increased the heat pain threshold^17^. Using a full-body illusion, Hänsel and colleagues found that seeing a mannequin’s body but not a non-corporeal object through an HMD increased the pressure pain thresholds and that the stronger the self-identification with the mannequin’s body, the higher the pain threshold^18^. Martini and colleagues showed that, in a virtual environment, the feeling of ownership over a virtual limb resulted in an increase in the heat pain threshold compared to seeing a non-corporeal object in either the virtual or real world^19^. These studies suggest an analgesic effect of viewing a fake/virtual body when it was felt to be one’s own in a healthy population.

Pain relief by viewing one’s own body may be extended to viewing a fake/virtual body felt as one’s own, as mentioned above. However, what happens to pain perception when one’s own body part is felt as not belonging to one anymore? The feeling of disowning one’s own body part can mainly be found in neuropsychological literature describing patients suffering from it (e.g. Refs.^20–22^, and see “Discussion” for details). Such an explicit feeling of disownership regarding one’s own body part has been argued to be a key aspect of a sense of bodily self, which is linked to the processing of bodily signals, as well as the feeling of body ownership^23,24^ because the sensory processing can result in either embodiment or disembodiment. It would be of great interest to shed light on the feeling of disownership to obtain a better understanding of not only clinical cases but also the nature of bodily self. Therefore, we designed an experimental paradigm to produce an illusion of disownership of one’s own limb, wherein participants observed the right-side view of their upper body from a third-person perspective (3PP) via an HMD. During the observation, they moved their left hand while keeping their left elbow behind the upper body, so that the connection of their arm to the torso was not visible, or in front of it, so they could see the arm being part of them. Several studies have investigated sensations related to disownership in healthy populations. The full-body illusion that makes participants have an illusory body^10,25^, a mannequin or another’s body^26–28^ at a camera position away from their real position reduces ownership on their body that they see in the field of view, in which this kind of disownership is induced by incongruence of visual-motor or visual-tactile stimuli. These studies involved disownership as the second effect of the body-ownership illusion presumably because most research has primarily focused on the fundamentals of the feeling of ownership over a foreign body. When participants view pre-recorded images of their own limb from a first-person perspective via an HMD, visual-tactile asynchrony elicits disownership directly on the limb^29–31^. Unlike these, we manipulated the spatial incongruence between the real hand’s position and the real hand’s visual feedback, that is visual-proprioceptive incongruence. The present study aimed to establish an induction of limb-disownership in healthy participants and to verify whether the feeling modulates pain perception using heat pain threshold measurements. We designed the experiment to test the hypothesis that spatial disparity between vision and proprioception of one’s limb induces limb-disownership. This hypothesis is supported by the rubber hand illusion studies reporting that such a spatial disparity between vision and proprioception reduces illusory body ownership^32–35^. Moreover, we hypothesized that the disownership feeling would have a modulatory effect on the pain evoked by heat stimuli delivered on participants’ real arm.

## Materials and methods

### Participants

Fifteen healthy volunteers (thirteen males, two females; mean age, 22.5 years; SD, 1.9) were recruited from among the students at Nagaoka University of Technology. We determined the sample size based on our previous study^36^, which used the same experimental setup and conditions as this study were used, and other studies of disownership^25,27,37^ and pain perception^16,18,38^. The power analysis was conducted using an alpha of 0.05, a power of 0.80, and a large effect size (*d* = 0.8) for a two-tailed test. The required sample size was determined to be fifteen. To match this, we recruited participants who have no previous participation in experiments investigating a bodily awareness. All participants had normal or corrected-to-normal vision, intact limbs, and no history of neurological or psychological disorders. All participants provided written informed consent to participate in the study after receiving an explanation of the study procedures. They were blind to the hypothesis tested by the study. The study was conducted according to the principles of the Declaration of Helsinki and was approved by the Ethics Committee of Nagaoka University of Technology. Participants received 1000 JPY for taking part in the study.

### Experimental setup and conditions

**Figure 1:**
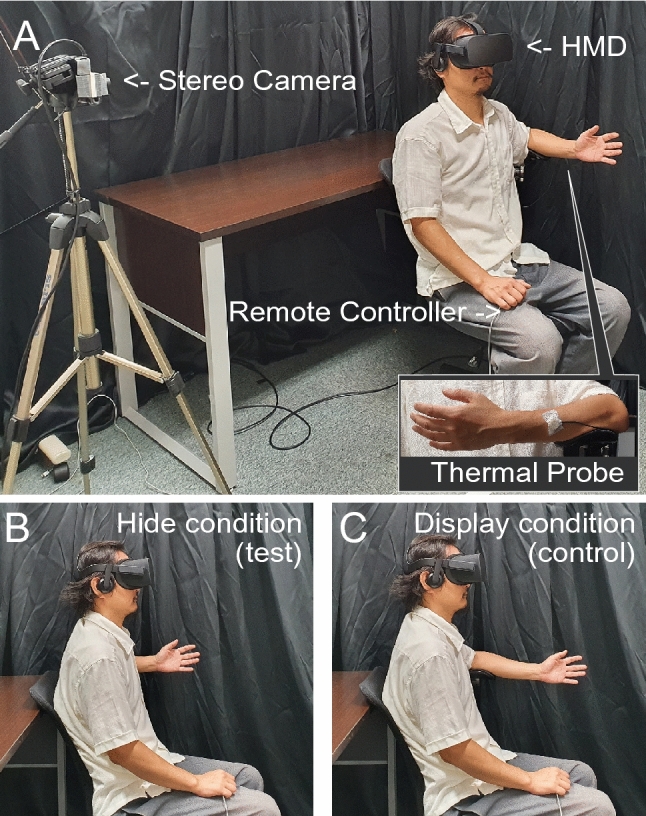
Experimental setup and view conditions. (**A**) The participant was fitted with a head mounted display, a thermal probe, and remote control. They observed themself from the right side via the display on which real time images were projected from a stereo camera. (**B**) In the test condition, represented by “Hide,” they opened and closed their hand while keeping their elbow out of sight. (**C**) In the control condition, represented by “Display,” they performed the same movements but kept their elbow in sight. Note that these pictures were staged with one of the authors for illustrative purposes. Informed consent for publication was obtained from the author shown in this figure.

The technical setup followed the methods of our previous study^36^. The observation system consisted of a stereo-camera (Ovrvision, Shinobiya.com Japan), an HMD (Oculus Rift cv1, Oculus VR, LLC, U.S; a resolution of 1080 × 1200 pixels per eye, a horizontal field of view of 110°, and a refresh rate of 90 Hz), and the Unity game engine (Unity Technologies, San Francisco, CA, USA), which transmitted real-time images captured by the camera onto the HMD. In this system, participants sitting on a chair placed their left hands forward and observed themselves from the right side via the HMD while moving their left hands in real time (Fig. 1A). In the test condition (“Hide”, see Fig. 1B) to induce the disownership feeling on their left hands, participants kept their left elbows behind their upper body during the observation. They could, therefore, only look at their hands and part of their forearms. Hiding the upper-arm makes the upper limb appear disconnected from the body. In the control condition (“Display”, see Fig. 1C), they placed their elbows in front of their bodies and could look at almost their whole upper limbs.

### Procedure

**Figure 2:**
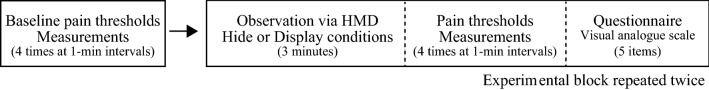
Experimental procedure. After pretesting to measure the baseline pain thresholds, we performed the experimental block in both conditions in random order (within-subject design).

Before starting the experiments, we conducted pre-testing to measure baseline pain thresholds (Fig. 2). Participants sat on a chair with both hands on their laps and closed their eyes. A thermal probe was attached to the back of the left arm (measurement point, see a box in Fig. 1A) and a remote control used to stop an increase of the probe temperature was held with the right hand. Pain threshold was measured four times at one-minute intervals between measurements. The experiments consisted of two blocks corresponding to either experimental condition (Hide or Display). The sequence of blocks was randomized and counterbalanced across the subjects. Following the baseline measurement, participants wore the HMD and placed their elbows on an armrest at the height of their chests. The stereo camera adjusted to the level of their eyes was placed in a position 1 m away from them toward their right (Fig. 1A). Then, an observation phase lasted for three minutes under either the Hide or Display conditions, in which a part of the left upper-limb, from the shoulder to the elbow, was out of or within view, respectively. During the observation, participants were instructed to open and close their left palms along with the ticking of a digital metronome at 60 beats per minute. After each trial, they performed pain threshold measurements four times in the same way as the baseline measurement, but kept their eyes on the images of their left hands which were projected on the HMD. Finally, after the measurements, a questionnaire was administered to evaluate the participants’ subjective feelings during the observation phase. Five minutes of rest were allowed between blocks.

### Measurements

#### Pain threshold

To measure pain thresholds, we applied thermal heat stimuli to the center of the posterior region of the left forearm by means of a thermal stimulator (UDH-105, UNIQUE MEDICAL, Tokyo, Japan). A thermal probe with a diameter of 20 mm was attached to the skin using surgical tape. Pain thresholds were determined in accordance with the method of limits^39^. The probe temperature was increased from normal skin temperature (the initial probe temperature was set to 32 °C on the device) at 1 °C per second. The temperature at which participants perceived the stimulus as painful was recorded as the pain threshold. Participants were instructed to press a stop button on a remote control held in their right hands on their laps as soon as they felt the probe temperature to be painful. For safety reasons, the probe temperature rapidly decreased immediately after pushing the button and the maximal temperature was set at 50 °C in case of not detecting a stop signal. Note that, prior to this experiment, participants were sufficiently trained to use the equipment through thermal stimulation to the dorsum of the first metacarpal space of their left hands. Pain threshold values in each measurement (pre-experiments as a baseline, post-Hide condition and post-Display condition) were represented by the average of four responses.

#### Subjective ratings

To measure to what extent participants subjectively experienced the disruption of body-related awareness (“Ownership,” “Agency,” “Body image,” and “Somatic Sense” are defined below) on their real limbs during the observation, a questionnaire consisting of five statements adapted from existing literature^23,36,40^ was administered. The items (translated from Japanese) were as follows: (1) “I felt as though my hand was not my own.”; (2) “I felt as though my hand was an imitation.”; (3) “I felt as though my hand was out of my control.”; (4) “I felt as though my arm shrank.”; (5) “I felt as though the sensation in my hand was lost.” Items 1 and 2 reflect the direct and indirect assessments of limb-disownership, respectively, and their average ratings in each condition were used to represent the strength of disownership (“Ownership”). Item 3 reflected the disruption of a sense of agency (“Agency”). A sense of agency is the feeling of authorship over one’s own actions and of controlling their execution^41^ and is another crucial component of self-awareness as well as a sense of ownership^40^. Item 4 asked about the visual appearance of the limb to consider the distortion of the body image (“Body Image”) (See also Ref.^42^). Additionally, Item 5 asked whether the somatic sensation in the limb was disrupted (“Somatic Sense”). Participants indicated their responses on a visual analog scale with the seven guides arranged at equal intervals ranging from − 3 (strongly disagree) to + 3 (strongly agree). Note that all the questions asked for the degree of disruption; higher values for each item indicated higher ownership disruption (disownership), Agency disruption, Body Image disruption, and Somatic Sense disruption, respectively. The questionnaire items were presented in random order.

### Statistical analysis

The Shapiro–Wilk normality test showed that the subjective ratings did not follow a normal distribution (*p* < 0.001), but the pain threshold changes from the baseline values did (*p* = 0.54). Therefore, we used nonparametric tests to analyze the subjective ratings data and parametric tests to analyze the pain threshold data only. To test the disruption of body-related awareness, including disownership, Wilcoxon signed rank tests were performed for comparisons of the subjective ratings between conditions. As we hypothesized a lack of association among body-related awareness in the test condition, correlation analyses (Spearman’s rank correlation) were also conducted to investigate the possible relationships between ratings of body-related self-awareness in each experimental condition. To test the modulatory effects of disownership on pain perception, a one-sample *t*-test and a paired *t*-test were performed for comparison of the pain threshold changes from the baseline values within and between conditions. To explore how the disownership feeling affected pain threshold, Spearman’s rank correlation was performed, investigating the association between the subjective ratings and the pain threshold changes. Moreover, we classified participants into subjectively “Disowning” and “Owning” groups, based on whether they had positive or negative ratings in the disownership ratings for the test condition (“Hide”). To test whether participants who felt the explicit feeling of disownership responded differently to painful stimulation from the others, an independent *t*-test was then performed comparing the pain threshold between groups. The significance level was set at *p* < 0.05. Data were analyzed with statistics software (R, The R Foundation for Statistical Computing, Vienna, Austria).

## Results

### Subjective ratings for disruption of body-related awareness

**Figure 3:**
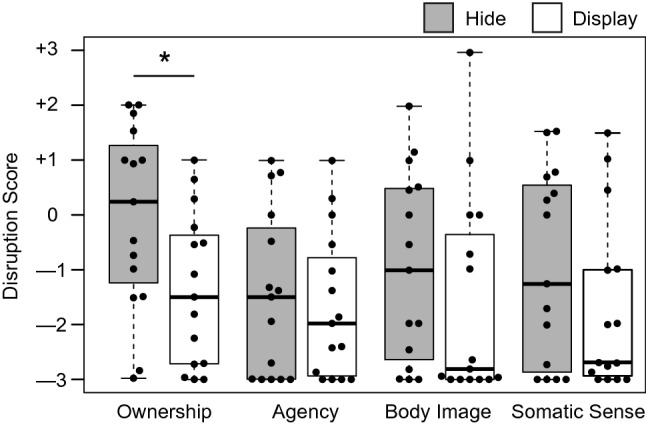
Subjective ratings for disruption of body-related awareness. Higher values indicates higher disruption scores for each item without reverse scoring because all the questions asked for the degree of disruption. Each data point represents a participant’s score. An asterisk indicates the statistical significance (*p < 0.05). Box-and-whisker plots represent the median of the data (thick lines), data between the first and third quartiles (boxes), and the maximum and minimum data (whiskers). There are no outliers.

Questionnaire items 1 and 2 concerned the participants’ assessments of limb-disownership. The difference scores between the conditions in item 1 were significantly correlated with those in item 2 (*r* = 0.67, *t*(13) = 3.43, *p* < 0.01). Therefore, we used their average ratings in each condition as the disownership scores (“Ownership”). The disownership scores varied significantly between conditions (*Z* = 2.17, *p* < 0.05, effect size *r* = 0.56), indicating a greater disownership feeling in the Hide condition (median = 0.24) compared to the Display condition (median = –1.50). Other items had no significant differences between conditions (“Agency”: *Z* = 0.76, *p* = 0.49; “Body Image”: *Z* = 1.41, *p* = 0.18; and “Somatic Sense”: *Z* = 1.25, *p* = 0.23). We found that the median scores were positive (above zero) only for the disownership question in the test condition (Fig. 3).

**Figure 4:**
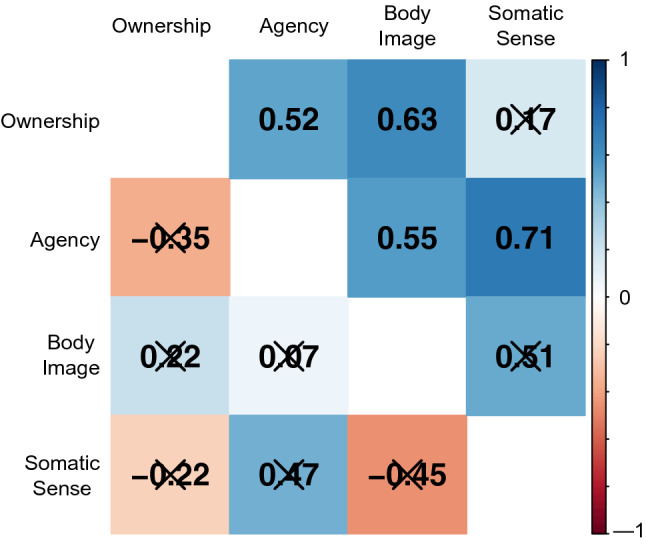
Correlation matrix between subjective ratings for disruption of body-related awareness. Upper triangle represents correlation coefficients in the Display condition and the lower part represents the Hide condition. X-marks on values indicate statistically non-significant correlations (*p* > 0.05).

In the Display condition, we observed significant correlations between ratings for disruptions of body-related self-awareness (Ownership vs. Agency: Spearman’s rho = 0.52, *p* < 0.05; Ownership vs. Body Image: Spearman’s rho = 0.63, *p* < 0.05; Agency vs. Body Image: Spearman’s rho = 0.55, *p* < 0.05; Agency vs. Somatic Sense: Spearman’s rho = 0.71, *p* < 0.01) except for two combinations with an item regarding somatic sensation (Ownership vs. Somatic Sense: Spearman’s rho = 0.17, *p* = 0.55; Body Image vs. Somatic Sense: Spearman’s rho = 0.51, *p* = 0.05). By contrast, there were no significant correlations between any combinations of the ratings in the Hide condition (Fig. 4).

### Relationship between disownership ratings and pain threshold changes

**Figure 5:**
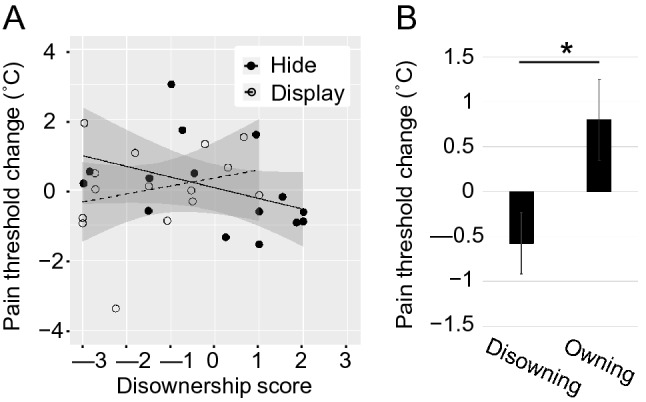
Pain threshold change from baseline measure. (**A**) Scatterplot showing the relationship between ownership disruption ratings and pain threshold change in the Hide (black circles, solid line) and Display (white circles, dashed line) conditions. Shaded area corresponds to 95% confidence intervals for the linear trends. (**B**) Averaged pain threshold changes of the two groups, where participants were classified into “Disowning” and “Owning” groups based on their disownership scores. Error bars represent the standard error. An asterisk indicates the statistical significance (*p < 0.05).

The pain threshold changes from the baseline had no significant differences between conditions (*t*(14) = 0.12, *p* = 0.90) or in each condition (Hide: *t*(14) = 0.20, *p* = 0.84, *M* = 0.07; Display: *t*(14) = 0.09, *p* = 0.93, *M* = 0.03). However, we found a significant negative correlation between the pain threshold changes and disownership ratings in the test condition (Spearman’s rho = –0.52, *p* < 0.05) but not in the control condition (Spearman’s rho = 0.27, *p* = 0.33. see Fig. 5A). Therefore, we assumed that participants who explicitly felt limb-disownership in the Hide condition might be more likely to experience decreased pain threshold. We thus separated participants into two groups based on whether their ratings for disownership were positive (“Disowning” group) or negative (“Owning” group). There were eight participants in the “Disowning” group and seven in the “Owning” group. Consequently, participants who experienced limb-disownership produced negative pain threshold changes (*M* = − 0.58), while those who did not experience limb-disownership produced positive changes (*M* = 0.8) (Fig. 5B). Pain threshold changes varied significantly between groups in the Hide condition but not in the Display condition (Hide: *t*(13) = 2.46, *p* < 0.05, Hedges’*g* = 1.12; Display:* t*(13) = 0.88, *p* = 0.40).

## Discussion

We developed a paradigm to induce the feeling of one’s limb as not belonging to oneself and investigated the modulatory effects of the disownership feeling on pain perception. Participants observed themselves from 3PP via an HMD in real-time while the upper limb appeared disconnected from the upper body despite the fact that participants, following instructions, just occluded their elbow behind the upper body. We demonstrated that this paradigm elicits a disownership illusion; participants rated “disownership” over their own hand significantly higher than in the control condition. The novelty in this paradigm is in the method we used to induce disownership. We created a spatial disparity between the visual feedback and the proprioception of their left upper limb, that is visual-proprioceptive incongruence rather than visual-tactile or visual-motor incongruences^10,25–30^. Newport and Gilpin showed that, using tricks making the hand seen from the first person perspective disappear, the spatial incongruence between vision and proprioception causes multisensory disintegration^37^; they did not investigate disownership specifically, and their paradigm is different in participants’ viewpoint from ours, but it is still in line with our findings.

Pain threshold changes had no difference between conditions, suggesting that the paradigm per se did not influence pain perception; this finding aligns with Mohan et al.’s reporting no pain relief with the rubber hand illusion^16^. Nevertheless, the degree of disownership in the test condition was negatively correlated with pain threshold changes. On closer inspection, we found that the participants who intensely felt limb disownership (the “Disowning” group) had lower pain thresholds comparing the participants who did not (the “Owning” group), suggesting those who are susceptible to body disownership manipulations were more sensitive to painful stimuli. Previous studies have reached inconsistent conclusions on whether the ownership illusion (e.g., the rubber hand illusion) increases^17–19^ or has no effect on^16^ the pain threshold. Hence, our disownership creation method provides a novel perspective on the relationship between the (dis)ownership feeling and pain perception. While multisensory (dis)integration causing (dis)ownership might not necessarily affect pain perception, the sense of bodily self at the individual level might associated with pain perception.

The disownership feeling is not just an absence of the ownership feeling; it is explicitly perceiving the lack of ownership of one’s own body or the disruption of self-specific embodiment^24^. Some researchers have suggested that the rubber hand illusion is accompanied by implicit loss of ownership toward participant’s own unseen hand while inducing an ownership feeling over the seen fake hand^40,43^; however, this does not necessarily indicate an induction of disownership^23^. Rather, there are several clinical conditions illustrating the phenomenology of the disownership feeling. Patients suffering from depersonalization often do not feel that their own bodies belong to them and are obsessed with reassuring themselves of its existence by touching and even injuring their bodies^20^. Somatoparaphrenia leads patients to experience the loss of limb ownership, often misattributing it to somebody else^21^. People with body integrity dysphoria (BID) deny the ownership of their own limbs and desire amputation to make them complete^22^.

In a healthy population, the feeling that “my body is mine” is taken for granted and being conscious of body ownership, let alone disownership, is rare. Body ownership can be felt often when body-related information becomes unreliable. Therefore, creating an unreliable situation allows us to experimentally manipulate body ownership^44^. Indeed, the rubber hand illusion paradigm leads participants to detect multisensory conflict while observing the obviously fake body part, and consequently to feel the ownership of the proxy^45,46^. Meanwhile, our approach leads participants to detect their unseen upper-arm while seeing their body; consequently, they feel the disownership of their own limb. In other words, prohibiting participants from confirming the connection between their limb and body segregateds and disintegrates visual and proprioceptive signals; after that participants feel the exclusion of the limb from their body. Our results of subjective ratings corroborate that participants explicitly felt disownership feelings of their own limbs. Additionally, according to the open question after the experiments, one participant, who rated disownership at the highest score, expressed that they saw the hand as someone else’s. Another, who rated disownership at the third highest score, showed discomfort toward the hand they saw. It is interesting that these spontaneous reports remind us of patients’ complaints following disownership feeling (namely alienation^21^ and unpleasantness^22^, as mentioned above).

Loss of correlations among ratings regarding body-related self-awareness also underpins that the limb-disownership feeling was induced in our experiments. Body-related self-awareness has been defined as the feeling that conscious experiences are bound to the self and are experiences of a unitary entity^47–49^. It involves several aspects such as a sense of body ownership, a sense of agency over one’s actions, feeling the body in space, and the perception of external/internal signals on the body^40^. In particular, it has been demonstrated that moving the embodied rubber hand results in a significant correlation between the subjective ratings of agency and ownership^50^. Likewise, we found a significant correlation between ownership and agency ratings in the control condition but not in the test condition. Moreover, our study showed that the correlation between every combination of body-related self-awareness (ownership, agency, body image, and somatic sensation) disappeared in the test condition. Our experimental manipulation could successfully disrupt bodily self-awareness as the experience of a unitary entity and elicit the disownership feeling.

That we found no significant changes in pain threshold in or between our experimental conditions aligns with Mohan et al.’s^16^ conclusion that the rubber hand illusion did not affect pain perception. Our study’s conditions modulated multisensory integration by manipulating visual-proprioceptive congruence. The lack of integration between vision and proprioception might have led to the subjective disownership feeling but not to pain modulation. Nonetheless, our results showing a correlation between pain thresholds and the subjective ratings of disownership demonstrated that the higher the subjective feelings of limb-disownership, the lower the pain threshold, and thus, the higher the sensitivity to painful stimuli. This contrasts with some previous studies reporting that the sense of ownership relieves pain. What does the low pain threshold reflect? Martini and colleagues showed that the subjective ratings of owning a transparent virtual arm are negatively correlated with heat pain threshold, suggesting a high sensitivity to painful stimuli when seeing the virtual body that visually fades away^51^ (for the opposite see Ref.^52^). Authors speculate that the general reduction of pain threshold could function as an alert system since the blurred boundary of one’s own body decreases the predictability of potential damage. Likewise, limb-disownership feelings in this study might activate protective mechanisms to compensate for the uncertainty regarding one’s own limb. Becoming susceptible to external stimuli may contribute to recovering one’s own limb that is temporarily lost because of the illusion. The unusual disownership feeling in healthy participants is likely to cause a reduced pain threshold to maintain a body image that has been accumulated over a long period of time. Interestingly, pain responses of patients who feel limb-disownership is opposite to our healthy participants. A recent study reported that patients with BID demonstrated higher pain thresholds when thermal stimulation was applied to the affected leg (felt disowned) compared to the unaffected leg (felt owned)^53^. For patients with BID, the affected body part has been excluded from their body image. Therefore, the patients can hardly perceive pain on the affected limb since protective mechanisms originally fail to function. Moreover, the idea that the feeling of disownership leads to becoming susceptible to pain for the maintenance of a body image may provide new insights in previous studies. A low heat-pain threshold was observed when seeing an apparently injured rubber hand^54^ and when seeing a virtual arm with reddish skin reminiscent of inflamed, hot, and more sensitive skin^55^. Additionally, a vision of potentially painful stimuli, the rubber hand being pricked by a sharp knife, decreases the mechanical pain threshold^56^. Such undesirable features of the fake body would have urged participants to refuse the ownership feeling over the proxy limb to maintain their intact body images when painful stimulation is delivered. The participants may feel disownership over the proxy at the moment of perceiving pain.

The paradigm in this study inducing limb-disownership needs further research to place it in the context of conventional embodiment studies^7^. Our questionnaire was adapted from previous literature^23,40^ to address the disruption of body-related self-awareness. While directly asking about explicit feelings is needed to grasp participants’ experiences, cognitive biases may affect subjective measures, such as demand characteristics^57^ and the anchoring effect^58^. However, some studies have reported that such biases were negligible in the rubber hand illusion^59^. To improve understanding of the phenomenology of body-disownership feelings and the relationship between subjective feeling and pain perception at the individual level, future studies must investigate the effects of such biases and develop questionnaire items by adding control statements. Furthermore, physiological reactions to the disownership feeling should be investigated. It has been reported that changes in the experience of ownership over a fake/virtual body often result in physiological changes such as a decrease in skin temperature^43,60^ (but see Ref.^61^) and an increase in skin conductance responses to stimuli threatening the proxy^26,62^. By contrast, the reduced threat-evoked skin conductance responses have been reported in the previous disownership-related studies^25,27–29^. Further studies may follow these protocols to link our paradigm with previous embodiment paradigms.

## Data Availability

All data are available from the corresponding author upon reasonable request.
